# An Evaluation of the Use of Aggressive Fluid Resuscitation in the Early Treatment of Sepsis Patients

**DOI:** 10.7759/cureus.13518

**Published:** 2021-02-23

**Authors:** William N Payne, Alfred Tager, Mike Broce, Dany Tager, Marion Hoy, Hythem Abad

**Affiliations:** 1 Emergency Medicine, Charleston Area Medical Center, Charleston, USA; 2 CAMC Education and Research Institute, Charleston Area Medical Center, Charleston, USA; 3 Internal Medicine, Charleston Area Medical Center, Charleston, USA

**Keywords:** chf exacerbation, sepsis, congestive heart failure, fluid resuscitation, sepsis treatment

## Abstract

Introduction

Fluid resuscitation is a critical aspect of the sepsis protocol with the usual initial dose being 30 mL per kilogram. Although this dose is well accepted in patients with normal cardiac function, there is some significant variation in clinical practice concerning the optimal fluid resuscitation in septic patients with underlying congestive heart failure (CHF). Many different approaches have been tried to best treat these patients by using lesser volumes of fluid. The purpose of this retrospective study is to attempt to better define optimal fluid resuscitation in congestive heart failure patients and whether standard fluid resuscitation exacerbates CHF in these cases.

Methods

This was a retrospective study involving patients admitted to the Emergency Department (ED) during the time period of September of 2016 through March of 2019 with a primary diagnosis of sepsis and pre-existing CHF. Data collected from the data warehouse and patient charts included demographics, total amount of fluid received in the ED and outcome data. Evidence of fluid overload (chest X-ray [CXR] evidence, rising B-type natriuretic peptide [BNP], or use of diuretics), was evaluated with respect to in-hospital mortality, white blood cell (WBC) count and comorbidities (chronic obstructive pulmonary disease [COPD], hypertension and coronary artery disease).

Results

There were 422 patients included in the cohort. Of the 422, 113 (26.8%) patients showed evidence of fluid overload on CXR during hospital stay and received diuretics and therefore considered in the CHF exacerbation group. The patients that experienced CHF exacerbation were significantly older (mean ± SD, 70.9 ± 11.8 years versus 67.4 ± 15.1 years, p=0.014). Patients with exacerbation also received more fluid (median and interquartile range, 3.0, 2:5.5 L versus 2.0, 1:4.3 L, p=0.017). The receiver operating characteristic curve analysis for fluid to predict exacerbation resulted in an area under the curve of 0.59 with a 95% confidence interval (CI) of 0.52 to 0.65, p=0.012. The Youden Index was used to determine an optimal cutoff value of 2.6 L. The percentage of patients in the exacerbation group above the threshold was significantly higher (57.3%) than those without exacerbation (43.3%), p=0.019. Following multivariate analysis, age greater than 60 (odds ratio [OR]: 2.5; CI: 1.4-4.6, p=0.003) and fluid cutoff of 2.6 L (OR: 1.9; CI: 1.2-3.1, p=0.007) were both found to be independent predictors of CHF exacerbation. There was no significant difference in mortality based on the total fluid received in the ED.

Conclusion

The findings of this study showed that septic patients with pre-existing CHF who received more than 2.6 L of fluid in the ED were 90% more likely to develop symptoms of CHF exacerbation with no evidence of lowering mortality compared to the group that received less than 2.6 L. Our data supports the practice of limiting total fluid resuscitation in CHF to 2.6 L and reconfirms the idea that fluid resuscitation for patients with CHF needs to be individualized.

## Introduction

Although improving, sepsis mortality remains high. Refining the approach to sepsis treatment and making it more individualized in some groups may further improve morbidity and mortality. The aggressive use of fluids is a major aspect of the sepsis protocol to maintain tissue perfusion by preventing hypovolemia. The usual dose of crystalloid is 30 mL per kilogram. Fluid resuscitation is just one aspect of sepsis resuscitation in addition to antibiotics, vasopressors, and other interventions. Optimal fluid resuscitation of patients with congestive heart failure (CHF) with reduced ejection fraction (HFrEF) has been less well established due to the risk of precipitating or exacerbating an episode of HFrEF. The acceptance of 30 mL/kilogram has been shown to be consistently used by 89% of physicians in patients with normal heart function, but the same survey found 39% of practitioners used lesser volumes for fluid resuscitation in CHF patients [1]. This raises concerns about the basis of this wide variability in practice [2]. The first question concerns whether the variation from the 30 mL/kg protocol causes worse outcomes in patients with CHF. Numerous studies have measured outcomes using various parameters often with no superiority and sometimes demonstrated undesirable effects of full fluid resuscitation. In addition to pre-existing heart disease, septic patients may demonstrate elevated troponin I values due to demand ischemia [3]. No difference in mortality was found in a recent study comparing standard fluid resuscitation in HFrEF with the use of 1000 ml less IV fluid over the first 24 hours [4]. Fluid overload may cause tissue edema in septic patients due to increased vascular permeability and other factors [5]. The second question concerns prompt identification of these patients in the emergency setting and an objective basis for determining the volume of immediate fluid resuscitation. New data concerning the response of these HFrEF patients to fluids might help to better define an objective approach to treatment. This retrospective study examined outcomes in septic patients with HFrEF with respect to both overall outcomes and incidence of CHF exacerbations. The goal was to develop criteria for a more precise use of fluid resuscitation in patients who have some degree of impairment in their cardiac function.

## Materials and methods

This is a retrospective study involving patients admitted to the Emergency Department (ED) during the time period of September 2016 through March 2019 with a primary diagnosis of sepsis and pre-existing congestive heart failure with reduced ejection fraction. Data collected from the data warehouse and patient charts included demographics, the total amount of fluid received in the ED, and outcome data. Evidence of fluid overload such as chest X-ray (CXR) findings, rising B-type natriuretic peptide (BNP), or requiring the use of diuretics was evaluated with respect to in-hospital mortality; also considered were white blood cell (WBC) count and comorbidities such as chronic obstructive pulmonary disease (COPD), hypertension, and coronary artery disease. This study was approved by our local institutional review board. All patients had continuous telemetry of vital signs including heart rate, blood pressure, and pulse oximetry; cardiac rhythm was also continuously monitored by telemetry.

Statistical analysis

All analyses were performed using IBM SPSS Statistics for Windows, version 19.0 (IBM Corp., Armonk, NY). Descriptive statistics are expressed in terms of frequencies, percentages, or means ± one standard deviation (SD). Categorical variables were tested by chi-square or Fisher exact tests, and continuous variables were tested by two-sample t-test or Mann-Whitney U test where deemed appropriate. A ‘p’ value of 0.05 or less was considered significant. A receiver operating characteristic (ROC) curve was used to analyze and determine the cutoff point for the amount of fluid given to predict heart failure exacerbations. The area under the curve (AUC) was used to measure the performance of the ROC curve analysis, and the Youden Index [6] was used to determine the optimal cutoff point for best predicting exacerbation. Logistic regression analysis was performed to find predictors of CHF exacerbation in the study cohort. Variables identified by univariate analyses to approach significance, p<0.1, were included in a full multivariate logistic regression model, while backward stepwise regression was used until only the significant predictors of exacerbation remained.

## Results

There were 422 patients included in the HFrEF cohort. There were 224 males (53.1%) and 198 (46.9%) females with an overall average age of 68.3 ± 14.4 years. Of the 422, 113 (26.8%) patients showed evidence of fluid overload on CXR during the hospital stay and received diuretics and therefore considered in the HFrEF exacerbation group. The patients who experienced HFrEF exacerbation were significantly older (mean ± SD, 70.9 ± 11.8 years versus 67.4 ± 15.1 years, p=0.014). There were more patients with an age greater than 60 in the exacerbation group as well (81.4% versus 71.5%, p=0.044). Patients with exacerbation also received more fluid (median and interquartile range, 3.0, 2:5.5 L versus 2.0, 1:4.3 L, p=0.017) (Table 1). The ROC curve analysis for the amount of fluid to predict exacerbation resulted in an AUC of 0.59 with a 95% confidence interval (CI) of 0.52 to 0.65, p=0.012. The Youden Index was used to determine an optimal cutoff value of 2.6 L. The percentage of patients in the exacerbation group above the threshold was significantly higher (57.3%) than those without exacerbation (43.3%), p=0.019. Following multivariate analysis, age greater than 60 (odds ratio [OR]: 2.5; CI: 1.4-4.6, p=0.003) and fluid cutoff of 2.6 L (OR: 1.9; CI: 1.2-3.1, p=0.007) were both found to be independent predictors of CHF exacerbation. There was no significant difference in mortality based on the total fluid received in the ED. The mortality rate for those below the 2.6 L threshold was 15.0%, and 16.7% for those above the threshold, p=0.665. The mortality rate for those who experienced CHF exacerbation (16.8%) was similar to those who did not (15.2%), p=0.762 (Table 2).

**Table 1:** Patient demographics CAD, coronary artery disease; WBC, white blood cell; COPD, chronic obstructive pulmonary disease; CHF, congestive heart failure *Median and 25th-75th quartiles are reported. **A total of 67 patients did not receive fluid in the Emergency Department.

Variable	No CHF		CHF		p value
	Exacerbation (n=309)		Exacerbation (n=113)		
	n (mean)	% (SD)	n (mean)	% (SD)	
Age	(67.4)	(15.1)	(70.9)	(11.8)	0.014
Age > 60	221	71.5	92	81.4	0.044
Female	150	48.5	48	42.5	0.273
Hypertension	195	63.1	69	61.1	0.734
COPD	107	34.6	32	28.3	0.243
CAD	132	42.7	45	39.8	0.656
Total fluid*^,^**	(2.0)	[1, 4.3]	(3.0)	[2, 5.5]	0.017
Fluid cutoff (≥2.6 L)	109	35.3	59	52.2	0.019
WBC > 10,000	50	16.2	21	18.6	0.559

**Table 2:** Mortality outcome for patients CHF, congestive heart failure

Variable	No CHF		CHF		p value
	Exacerbation (n=309)		Exacerbation (n=113)		
	n (mean)	% (SD)	n (mean)	% (SD)	
Mortality	47	15.2	19	16.8	0.762
	Fluid cutoff		Fluid cutoff		
	<2.6 L, n=187		≥2.6 L, n=168		
Mortality	28	15.0	28	16.7	0.665

## Discussion

This study does indicate that there is a definite correlation between the use of increased amounts of IV fluids greater than 2.6 L in patients with underlying HRrEF with the development of complications of fluid overload necessitating the use of intravenous diuretics. The OR for this was 1.9. There was no increase in mortality in any of the subgroups. These results may more accurately define ideal fluid goals in congestive heart failure patients who are at greater risk of sepsis. The mortality from sepsis in patients with HFrEF is greater than that for those with normal ventricular function and could be related to demand cardiac ischemia compromising already impaired ventricular function [7]. Sepsis causes a greater percentage of death in patients with mild to moderate HFrEF compared to severe CHF in which direct cardiac causes predominate [8]. Our results provide evidence that 26 mL/kg of fluid provides a more precise treatment goal in HFrEF. Results of this study also corroborate results of other studies cited in this paper that show no detrimental effects of reduced fluids in HFrEF.

Limitation

This study has the obvious limitation of being a retrospective study. The study population reflects the demographic features of the local population, which is overall older with an increased incidence of hypertension, coronary artery disease, and chronic obstructive pulmonary disease. Such a population is obviously not typical of some other areas of the world. The study could also be repeated using a larger sampling of patients with consideration of more parameters including additional diseases such as diabetes. An additional study might also include hospital course, duration of stay, and discharge disposition.

## Conclusions

Study findings showed that septic patients with pre-existing HFrEF who received more than 2.6 L of fluid in the ED were 90% more likely to develop symptoms of HFrEF exacerbation with no evidence of lowering mortality compared to the group that received less than 2.6 L. The study suggests cautious use of crystalloid fluid in this group of patients, especially the ones older than 60 years. Our data supports the idea of limiting total initial fluid resuscitation in HFrEF to 2.6 L. This study reconfirms the idea that fluid resuscitation for patients with some degree of congestive heart failure needs to be individualized. One way of doing this is to consider something that can be done quickly at the bedside such as elevation of lower extremities to see if this improves vital signs in these patients. This would yield rapidly obtained bedside evidence to help guide fluid resuscitation and does not always require more comprehensive cardiac studies such as invasive monitoring or obtaining a formal echocardiogram that may not always be immediately available in the ED. Bedside echocardiography and lung ultrasound by emergency physicians and intensivists would be obviously useful but might be susceptible to some degree of operator variation.
